# The Potential Protective Effect of Curcumin on Amyloid-*β*-42 Induced Cytotoxicity in HT-22 Cells

**DOI:** 10.1155/2018/8134902

**Published:** 2018-01-15

**Authors:** Lu Zhang, Yu Fang, Xuan Cheng, Yajun Lian, Zhaoshu Zeng, Chuanjie Wu, Hongcan Zhu, Hongliang Xu

**Affiliations:** ^1^Department of Neurology, The First Affiliated Hospital of Zhengzhou University, Zhengzhou, Henan, China; ^2^Department of Intensive Care Unit, The First Affiliated Hospital of Zhengzhou University, Zhengzhou, Henan, China; ^3^Department of Legal Medicine, The College of Basic Medical Sciences, Zhengzhou University, Zhengzhou, Henan, China

## Abstract

**Background:**

We aimed to investigate the effect and mechanism of curcumin (CUR) in Alzheimer's disease (AD).

**Methods:**

Mouse hippocampal neuronal cell line HT-22 was treated with A*β*1–42 and/or CUR, and then cell viability was evaluated by cell counting kit 8, Beclin-l level was detected using western blotting, and the formation of autophagosomes was observed by transmission electron microscopy (TEM). Furthermore, transcriptome sequencing and analysis were performed in cells with A*β*1–42 alone or A*β*1–42 + CUR.

**Results:**

A*β*1–42 treatment significantly inhibited cell viability compared with untreated cells (*P* < 0.01). After treatment for 48 h, CUR remarkably promoted cell viability compared with cell treated with A*β*1–42 alone (*P* < 0.01). Compared with cells treated with A*β*1–42 alone, the expression of Beclin-1 was slightly reduced in cells with combined treatment of A*β*1–42 with CUR (*P* < 0.05). Consistently, TEM results showed that CUR inhibited the formation of autophagosomes in cells treated with A*β*1–42. Furthermore, the protein-protein interaction network showed five key genes, including MYC, Cdh1, Acaca, Egr1, and CCnd1, likely involved in CUR effects.

**Conclusions:**

CUR might have a potential neuroprotective effect by promoting cell viability in AD, which might be associated with cell autophagy. Furthermore, MYC, Cdh1, and Acaca might be involved in the progression of AD.

## 1. Introduction

Alzheimer's disease (AD), a progressive neurodegenerative disease, is most common type in senile dementia [1]. The morbidity of AD is increasing with the aging population, which endangers physical, psychological, and living quality of old people due to high fatality rate and disability rate [2]. Although a significant progress has been obtained in the pathogenesis of AD, the effective treatments to block the development of AD are unsatisfactory. Therefore, it is urgent to explore the pathogenesis of AD in depth and search for new therapeutic targets and drugs for AD.

Curcumin (CUR) is major polyphenol extracted from the rhizome of curry spice turmeric and is widespread traditional medicine in South and Southeast Asia [3]. Increasing evidences have demonstrated that CUR has the beneficial properties such as antitumor, antioxidant and anti-inflammatory [4–6]. Epidemiological studies have reported that the lowest prevalence rate of AD is found in India, which may be associated with common eating curry spice in India population [7, 8]. Previous study also has revealed that curry consumption is related to better cognitive functions in old people [9]. Several studies have further shown that CUR can significantly improve cognitive functions by reducing oxidative damage and inflammation and then inhibiting amyloid-*β*-protein (A*β*, especially A*β*-42) aggregation in the experimental AD models [10–12].

It has been well-known that A*β* is a main marker protein in the development of AD [13] and intracellular A*β*-42 aggregation is proved to play a key role in the early stage of AD [14]. Abnormal autophagy can lead to early neuropathic damage in AD [15], which involves the secretion of A*β* [16]. In the early stage of AD, autophagy can eliminate abnormal protein A*β* and has a neuroprotective effect in AD, while continued A*β* aggregation induces dysfunction of lysosomal degradation, which leads to the leakage of lysosomal proteins from autophagic vacuoles and the acidification of cytosol, eventually resulting in neuronal death in the late stage of AD [17]. Wang et al. [18] have reported that CUR induces autophagy by downregulating phosphoinositide 3-kinase (PI3K)/Akt/mammalian target of rapamycin (mTOR) signaling pathway and inhibiting the production of A*β* in APP/PS1 double transgenic mice. However, further studies are still necessary to investigate the underlying mechanisms of CUR in AD.

In the present study, mouse hippocampal neuronal cell line HT-22 was treated with A*β*1–42 and/or CUR, and then the cell viability, the expression of autophagy-related protein Beclin-l, and the formation of autophagosomes were detected in HT-22 cells. Furthermore, transcriptome sequencing was performed in cells with A*β*1–42 alone and cells with A*β*1–42 + CUR, respectively, and then the function enrichment and protein-protein interaction (PPT) analysis in differentially expressed genes (DEGs) were conducted, aiming to investigate the underlying mechanisms of CUR in AD.

## 2. Materials and Methods

### 2.1. Cell Culture

HT-22 cells were purchased from JENNIO Bio Technology Co., Ltd. (Guangzhou, China). Cells were cultured in Dulbecco's Modified Eagle Media (DMEM, Gibco Co., Ltd., Carlsbad, CA, USA) containing 10% fetal bovine serum (Gibco Co., Ltd.) and 1% penicillin/streptomycin (Gibco Co., Ltd.) in 37°C incubator with 5% CO_2_.

### 2.2. Detection of Cell Viability

HT-22 cells (1.0 × 10^4^ cells/well) were seeded into 96-well plates. The second day, the cells were treated with 5 *μ*M A*β*1–42 [oligomer, dissolved in dimethyl sulfoxide (DMSO) and incubated at 37°C for 72 h to induce aggregation, Sigma, Louis, MO, USA] [19], 5 *μ*M A*β*1–42 + 5 *μ*M CUR (dissolved in DMSO, Sigma), 5 *μ*M A*β*1–42 + 10 *μ*M CUR, and 5 *μ*M A*β*1–42 + 15 *μ*M CUR [20], respectively. HT-22 cells without any treatment served as control group. After incubation for 24 h and 48 h, respectively, cells in each well were incubated with 10 *μ*L cell counting kit 8 (CCK8, Dojindo Co., Ltd, Tokyo, Japan) for 2 h. Ultimately, absorbance was read at 450 nm using Synergy H4 microplate reader (BioTek, Winooski, VT, USA).

### 2.3. Western Blotting

The cells were treated with 5 *μ*M A*β*1–42 or 5 *μ*M A*β*1–42 + 10 *μ*M CUR for 48 h. The cells were collected and treated with RIPA buffer (Beyotime Institute of Biotechnology, Shanghai, China) on ice for 30 min. After centrifugation at 12,000 rpm for 15 min, supernatant was acquired and protein concentration was measured using the BCA Protein Quantitative Assay (Pierce, Rockford, IL, USA). Protein (30 *μ*g/lane) sample was separated and blotted to polyvinylidene fluoride membranes (Millipore, Belfor, MA, USA), which were blocked in 5% nonfat milk for 1 h. Then, the membranes were incubated with rabbit anti-mouse *β*-actin polyclonal antibody (1 : 1000, Proteintech, Chicago, IL, USA) or rabbit anti-mouse Beclin-l polyclonal antibody (1 : 500, Abcam, Cambridge, MA, USA) overnight at 4°C, followed by incubation with goat anti-rabbit IgG (H+L)-HRP (1 : 5000, Jackson, West Grove, PA, USA) for 2 h at room temperature. Proteins were expressed using enhanced chemiluminescence (ECL) kit (Millipore) and analyzed by Image J software. The results were quantified from three independent experiments.

### 2.4. Transmission Electron Microscopy (TEM)

The cells were treated with 5 *μ*M A*β*1–42 or 5 *μ*M A*β*1–42 + 10 *μ*M CUR for 48 h. Then, the formation of autophagosomes in cells was observed using TEM. Briefly, cells were collected and then fixed in 2.5% glutaraldehyde for 2 h at 25°C. After washing with phosphate buffered saline for 3 times, the cells were postfixed in 2% osmium tetroxide for 2 h and then dehydrated in graded alcohols. Subsequently, samples were sectioned and embedded in LX112 plastic. Finally, sections were stained with uranyl acetate and lead citrate, and electron micrographs were obtained by JEM-1230 TEM (JEOL, Japan).

### 2.5. Transcriptome Sequencing

The cells were treated with 5 *μ*M A*β*1–42 or 5 *μ*M A*β*1–42 + 10 *μ*M CUR for 48 h. Then, the cells were collected and the total RNA was extracted using Trizol (Invitrogen, Gaithersburg, MD, USA). The mRNA-seq library was constructed and then sequenced on the Illumina Genome Analyzer IIx sequencing platform. The raw reads were obtained by the Illumina instrument software and cleaned by removing reads with unknown bases “*N*” > 5%, adapter sequences, reads with more than 20%  *Q* < 20 bases, and reads with <30 bases. The clean reads were mapped to the mice reference genome based on NCBI by TopHat software. The gene expression values using fragments per kilobase of exon model per million reads were obtained by StringTie tool (V1.2.2) based on mice gene annotation.

### 2.6. Identification and Analysis of DEGs

DEGs between cells with A*β*1–42 samples and cells with A*β*1–42 + CUR samples were obtained using the Linear Model for Microarray package in R [21]. The cutoff criteria for DEGs were set up as follows: |log_2_  fold  change| value > 2 and the *P* value < 0.05. For functional analysis for DEGs, gene ontology terms (GO; http://www.geneontology.org) in biological process (BP) were performed based on the Database for Annotation, Visualization and Integrated Discovery [22]. In addition, PPI network for DEGs was constructed using the Search Tool for the Retrieval of Interacting Genes online database [23] and visualized using the Cytoscape [24] software.

### 2.7. Statistical Analysis

Statistical analysis was performed by SPSS 19.0 statistical analysis software (SPSS Inc., Chicago, IL, USA). Data were expressed as the mean ± SEM and analyzed by *t*-test. A value of *P* < 0.05 was considered significant and *P* < 0.01 was considered highly significant.

## 3. Results

### 3.1. Effect of CUR on Cell Viability in A*β*1–42 Treated HT-22 Cells

**Figure 1:**
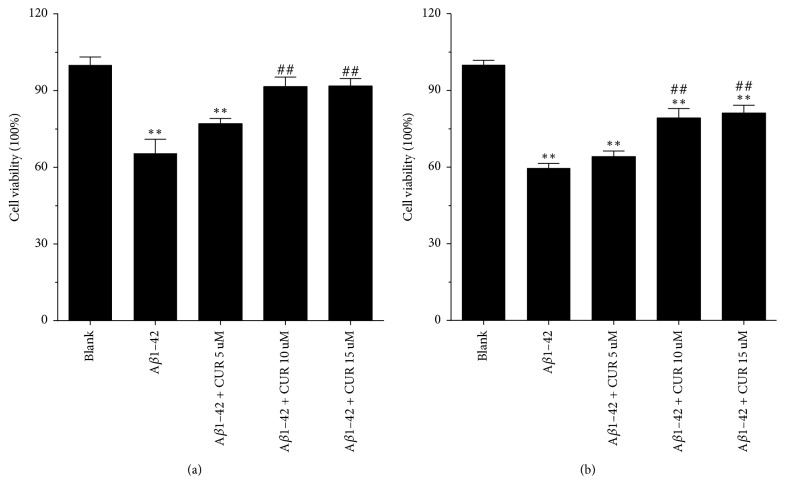
Curcumin (CUR) promoted cell viability in A*β*1–42 induced cells. Cell viability in untreated cells (blank), A*β*1–42 induced cells, and CUR + A*β*1–42 treated cells at 24 h (a) and 48 h (b) using CCK-8 assay. The experiment was repeated for three times. ^*∗∗*^*P* < 0.01 versus Blank group; ^##^*P* < 0.01 versus A*β*1–42 group.

CCK8 assay results showed that compared with untreated cells, cell viability was significantly inhibited in cells treated with A*β*1–42 alone (*P* < 0.01), while cell viability was remarkably increased after treatment with 10 *μ*M (*P* < 0.01) or 15 *μ*M (*P* < 0.01) but not 5 *μ*M CUR for 24 h (Figure 1(a)). Similarly, after treatment for 48 h, CUR (10, 15 *μ*M) significantly promoted cell viability in comparison with cell treated with A*β*1–42 alone (*P* < 0.01, Figure 1(b)). Based on CCK8 assay, the combined treatment with 5 *μ*M A*β*1–42 + 10 *μ*M CUR for 48 h was used in subsequent experiment.

### 3.2. Effect of CUR on Cell Autophagy in A*β*1–42 Treated HT-22 Cells

**Figure 2:**
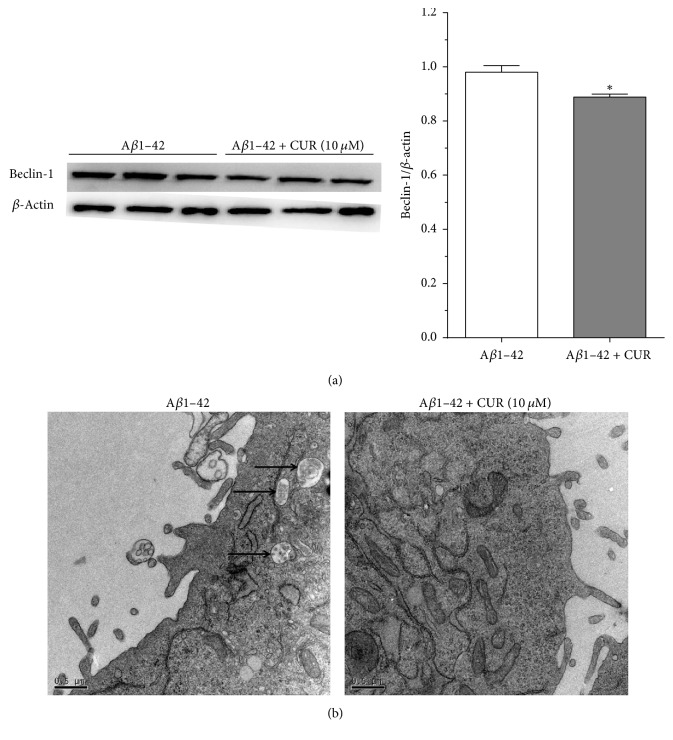
Curcumin (CUR) inhibited cell autophagy in A*β*1–42 induced cells after treatment for 48 h. (a) The protein expression of Beclin-1 in A*β*1–42 induced cells and CUR + A*β*1–42 treated cells using western blotting; (b) cell autophagosome in A*β*1–42 induced cells and CUR + A*β*1–42 treated cells using transmission electron microscopy. Bar = 0.5 *μ*m; ^*∗*^*P* < 0.05 versus A*β*1–42 group.

Western blotting results found that autophagy-related protein Beclin-1 was slightly downregulated in cells with combined treatment of A*β*1–42 and CUR compared with cells treated with A*β*1–42 alone (*P* < 0.05, Figure 2(a)). In addition, TEM results showed that autophagosome could be observed in cells treated with A*β*1–42 alone, while no autophagosome appeared in cells with combined treatment of A*β*1–42 and CUR (Figure 2(b)), which was consistent with the results of downregulated Beclin-1.

### 3.3. Function Enrichment Analysis of DEGs

Totally, 882 DEGs between cells with A*β*1–42 alone and cells with A*β*1–42 + CUR were obtained, including 324 upregulated DEGs and 558 downregulated DEGs. GOBP enrichment analysis showed that upregulated DEGs were significantly related to negative regulation of molecular function, epidermis development, metal ion transport, and keratinocyte differentiation, and downregulated DGEs were mainly correlative to intracellular organelle lumen, membrane-enclosed lumen, organelle lumen, nuclear lumen, and nucleolus. The top 10 GOBP terms with upregulated and downregulated DGEs are shown in Table 1.

### 3.4. PPI Analysis of DEGs

**Figure 3:**
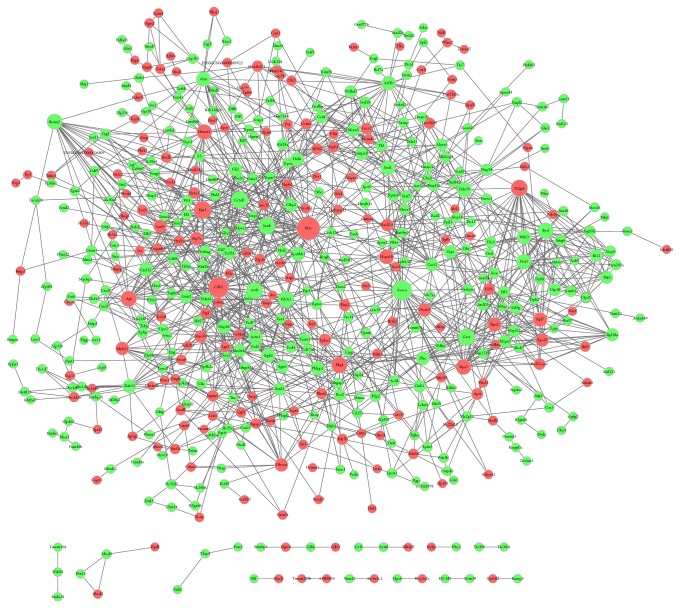
Protein-protein interaction network constructed for differentially expressed genes (DEGs). The red nodes stand for upregulated DEGs and the green nodes stand for downregulated DEGs.

Totally, 552 DEGs including 162 upregulated DEGs and 360 downregulated DEGs were involved in 1337 interaction pairs (Figure 3). There were 7450 edges in PPI network for DEGs (Figure 3).* MYC*,* Cdh1*,* Acaca*,* Egr1*, and* CCnd1* were located in the top 5 nodes with high degrees in PPI network.

## 4. Discussion

The present study found that CUR significantly promoted cell viability, reduced the expression of Beclin-1, and lowered the formation of autophagosomes in A*β*1–42 treated HT-22 cells. In addition, transcriptome sequencing results showed 324 upregulated DEGs and 558 downregulated DEGs, and PPI network showed that the pathogenesis of AD might be associated with* MYC*,* Cdh1*, and* Acaca* listed in the top 3 nodes with high degrees.

Previous study had shown a potential therapeutic role of CUR in the pathophysiology of AD [25]. Some* in vivo* studies demonstrated that oral administration of CUR could improve AD by removing A*β* deposition and improving behavioral impairment [10, 26]. It had been shown that CUR had an antiproliferation role in cancer cells [27]. However, this study found that CUR could promote cell proliferation. Similarly, Ma et al. [28] demonstrated that CUR could stimulate proliferation of rat neural stem cells. They found that low dose of CUR (0.1, 0.5, and 2.5 *μ*M) increased the proliferation of neural stem cells, whereas high doles of CUR (12.5 and 62.5 *μ*M) caused a decrease in the proliferation of neural stem cells [28], which was also consistent with our study. These results indicated the different role of CUR in cancer cells and neuronal cells. Autophagy had been reported to have contrary effect on A*β* aggregation in the different stage of AD [17]. In addition to antioxidant and anti-inflammatory effect, CUR could induce autophagy in various cancers, including human lung adenocarcinoma [29], colon cancer [30], glioblastomas [31], and oral cancer [32]. Furthermore, CUR was reported to induce autophagy and inhibit A*β* secretion in AD model mice [18]. Conversely, our study showed that CUR inhibited cell autophagy. This may explain that CUR removed intracellular A*β* depositions and then inhibited A*β*-induced toxicity, thereby exhibiting neuroprotective role by inhibiting cell autophagy [33]. However, our results showed only 10% inhibition of Beclin-1 expression caused by CUR treatment, so CUR-induced cell viability might be partly associated with cell autophagy in AD, while further study should be performed to confirm this ratiocination.

In order to further investigate the mechanism of CUR, transcriptome sequencing and bioinformation analysis were performed. The results found some important genes, such as* MYC*,* Cdh1*, and* Acaca* in PPI network.* MYC* oncogenes, containing C-myc, N-myc, and L-myc, had been proved to be overexpressed in tumor cells and closely associated with tumorigenesis by regulating cell proliferation, apoptosis, and differentiation [34]. In normal hematopoietic cells and hepatocytes, upregulated MYC expression could induce cell cycle progression [35, 36]. MYC was also overexpressed in AD and traumatic brain, which led to cognitive deficits and neurodegeneration [37, 38].* Cdh1 *gene was cell cycle-related gene and could activate anaphase-promoting complex (APC) [39]. Cdh1-APC had been demonstrated to control the G0 and G1 phases of the cell cycle and regulate axonal growth during the neuronal differentiation of the mammalian brain [40]. Cdh1 could promote neuronal survival and lead to apoptotic cell death by inhibiting cyclin B1 accumulation in primary cortical neurons, indicating that upregulated Cdh1 prevented neuron damage induced by the neurotoxicity of A*β* [41]. Similarly, the present study revealed that, in A*β*1–42 treated HT-22 cells, CUR increased the expression of MYC and promoted cell growth. Acetyl-CoA carboxylase *α* (ACC-*α*) protein, encoded by* Acaca *gene, was a key enzyme in fatty acid synthesis pathway and expressed in various cells especially in lipogenic tissues [42]. ACC-*α* had been reported to be a potential target in metabolic syndromes and cancers because of the roles in fatty acid metabolism [43]. Some studies had shown overexpressed ACC-*α* in some cancers, including breast cancer and prostate cancer, indicating the protective role for cancer cell survival [44–46]. Effective interventions against ACC-*α* had been reported to inhibit tumor growth by regulating cell fate, transformation, and differentiation [47]. However, some studies should be performed to investigate the effect of ACC-*α* on neurodegenerative disease. In addition, our study only suggested preliminary results and further experiments for the validation of DEGs expression were still needed.

## 5. Conclusions

The current study revealed that CUR might have a potential protective effect by promoting cell viability in AD, which might be associated with cell autophagy. Furthermore,* MYC*,* Cdh1*, and* Acaca* might be involved in the early stage of AD, which should be further confirmed.

## Figures and Tables

**Table 1:** The enriched pathways of DEGs between cells with A*β*1–42 alone and cells with A*β*1–42 + CUR.

DEGs	Terms	Name	Counts	Gene	*P* value
Upregulated DEGs	GO:0044092	Negative regulation of molecular function	7	ATP7A, MYC…	0.005451488
	GO:0008544	Epidermis development	6	ATP7A, GRPC5D…	0.018159092
	GO:0030001	Metal ion transport	12	ATP7A, MCOLN1…	0.019076392
	GO:0030216	Keratinocyte differentiation	4	GPRC5D, EVPL…	0.020403199
	GO:0052548	Regulation of endopeptidase activity	4	CDH1, MYC…	0.022720082
	GO:0043281	Regulation of caspase activity	4	CDH1, MYC…	0.022720082
	GO:0007398	Ectoderm development	6	ATP7A, GPRC5D…	0.023056172
	GO:0006812	Cation transport	13	ATP7A, MCOLN1…	0.023211511
	GO:0009913	Epidermal cell differentiation	4	GPRC5D, EVPL…	0.023930017
	GO:0052547	Regulation of peptidase activity	4	CDH1, MYC…	0.023930017

Downregulated DEGs	GO:0070013	Intracellular organelle lumen	40	SURF6, UTP18…	4.57*E* − 04
	GO:0031974	Membrane-enclosed lumen	41	HNRNPA2B1, SIRT4…	4.70*E* − 04
	GO:0043233	Organelle lumen	40	SURF6, UTP18…	4.81*E* − 04
	GO:0031981	Nuclear lumen	31	SURF6, UTP18…	0.002647478
	GO:0005730	Nucleolus	15	TSEN54, TBL3…	0.003596227
	GO:0005643	Nuclear pore	6	CSE1L, KPNA6…	0.006174947
	GO:0005739	Mitochondrion	39	PGS1, PDP2…	0.012109477
	GO:0046930	Pore complex	6	SIRT4, ACACA…	0.014833322
	GO:0005929	Cilium	8	TTC30B, TTC30A1…	0.018005139
	GO:0005912	Adherens junction	7	FMN1, ARHGAP31…	0.01885244
